# RNAi-Mediated Silencing of Paramyosin Expression in *Trichinella spiralis* Results in Impaired Viability of the Parasite

**DOI:** 10.1371/journal.pone.0049913

**Published:** 2012-11-21

**Authors:** Xiaoqin Chen, Yaping Yang, Jing Yang, Zhifei Zhang, Xinping Zhu

**Affiliations:** Department of Parasitology, School of Basic Medical Sciences, Capital Medical University, Beijing, China; New England Biolabs, United States of America

## Abstract

**Background:**

*Trichinella spiralis* expresses paramyosin (*Ts*-PMY) not only as a structural protein but also as an immunomodulatory protein to protect the worm from being attacked by host complement components. In this study, the functions of PMY in the viability and the growth development of *T. spiralis* were confirmed at the first time by silencing the gene function with RNA interference technique.

**Methods and Findings:**

To understand its functions in the viability of the worm, we used RNA interference to silence the expression of *Ts-pmy* mRNA and protein in the parasite. Significant silencing of *Ts-pmy* mRNA expression in larval and adult *T. spiralis* was achieved by siRNA and dsRNA through soaking and electroporation. Electroporation of *T. spiralis* larvae with 8 µM siRNA1743 or 100 ng/µl dsRNA-PF3 resulted in 66.3% and 60.4% decrease in *Ts-pmy* transcript and 52.0% and 64.7% decrease in *Ts*-PMY protein expression, respectively, compared with larvae treated with irrelevant control siRNA or dsRNA. Larvae treated with siRNA1743 displayed significant reduction in molting (40.8%) and serious surface damage as detected with SYTOX fluorescent staining. Infection of mice with larvae electroporated with *Ts-pmy* siRNA1743 resulted in 37.6% decrease in adult worm burden and 23.2% decrease in muscle larvae burden compared with mice infected with control siRNA-treated larvae. In addition, adult worms recovered from mice infected with siRNA-treated larvae released 24.8% less newborn larvae.

**Conclusion:**

It is the first time RNAi was used on *T. spiralis* to demonstrate that silencing PMY expression in *T. spiralis* significantly reduces the parasite’s viability and infectivity, further confirming that *Ts-*PMY plays an important role in the survival of *T. spiralis* and therefore is a promising target for vaccine development.

## Introduction

Trichinellosis, a widespread foodborne zoonosis, is acquired through the ingestion of undercooked meat containing encapsulated larvae of *Trichinella spiralis* (*T. spiralis*) [1]. The *Trichinella* parasite is an intestinal nematode that infects more than 100 mammalian species, including humans [2]–[3]. Human trichinellosis outbreaks occur in many parts of the world, and it has been regarded as a reemerging disease [4]–[6]. Trichinellosis is not only a public health hazard that affects approximately 11 million people worldwide [4] but also represents an economic problem in porcine animal production and food safety [2]. It is difficult to prevent and control *Trichinella* infection because of the wide distribution of domestic and wild animal reservoirs and the lack of specific clinical symptoms or signs for diagnosis [6]–[8]. These concerns have prompted interest in identifying molecules that play important roles in the establishment of infection in domestic animals and humans as targets for developing therapeutic and preventive vaccines [9]–[10].

In an effort to identify protective antigens for vaccine development against *Trichinella* infection, we have identified paramyosin of *T. spiralis* (*Ts*-PMY) as not only a structural protein of the worm [11]–[12] but also an immunomodulatory protein expressed on the surface of larvae and adult worms that plays an important role in the immune escape from host complement components [13]–[14]. In our previous reports, a full-length cDNA encoding *Ts*-PMY was cloned by immunoscreening a *T. spiralis* cDNA library with immune sera, and the recombinant *Ts*-PMY was found to elicit partial protective immunity in BALB/c mice against *T. spiralis* larval challenge [15]–[16]. Additionally, Zhang et al demonstrated that PMY was constitutively expressed in all of the developmental stages of *T. spiralis* and bound to human complement C8 and C9 to inhibit complement-mediated killing of the parasite [17]. The protective epitope of *Ts*-PMY has been identified at the N-terminus of the protein, and it elicited even better protection in a vaccine trial compared with the full-length protein [18]. Despite its promise as a vaccine against *T. spiralis* infection, the exact function of PMY in the establishment of parasitism in the host is still not well understood, except for its binding activities to human complement C8 and C9. We therefore employed RNA interference (RNAi)-based gene silencing to explore the biological functions of PMY in *T. spiralis* through a loss-of-function approach.

In this study we examined the feasibility of using RNAi in the larvae and adult worms of *T. spiralis* for the first time and demonstrated that the silencing of *Ts*-*pmy* in *Trichinella* induced by small interfering RNA (siRNA) or double-stranded RNA (dsRNA) resulted in significant reduction in *Ts*-PMY expression at both RNA transcript and protein levels and the impaired viability of the parasite in both *in vitro* culture and *in vivo* infection.

## Materials and Methods

### Ethics Statement

All experimental animals were purchased from Laboratory Animal Services Center of Capital Medical University (Beijing, China). All experimental procedures were reviewed and approved by the Capital Medical University Animal Care and Use Committee and were consistent with the NIH Guidelines for the Care and Use of Laboratory Animals.

### Parasites

*T. spiralis* (ISS 533) parasites used in this study were maintained in ICR mice. Each mouse was orally infected with 400 *T. spiralis* larvae. Adult worms were collected from the intestines of mice [19], and muscle larvae were recovered from the muscle of infected mice using a standard pepsin–hydrochloric acid digestion method [20]. Crude somatic extracts of the different stages of *T. spiralis* were prepared by conventional methods [21], and the protein concentration was determined by the BCA assay (Pierce, US).

### Preparation of siRNA

The full-length cDNA encoding *Ts*-PMY (GenBank accession# EF429310) was used to design for the siRNA sequences with BLOCK-iT RNAi Designer (http://www.invitrogen.com/rnaidesigner). The *Ts-pmy*-specific 25-mer siRNA oligos (Stealth ™ RNAi duplexes) were chemically synthesized by Invitrogen (Life Technologies, US). The sequences of the three specific siRNA oligos and corresponding Stealth control siRNA used in this study are shown in Table 1. The same control siRNA labeled with FITC (Life Technologies, US) was used to monitor the transfection efficiency.

**Table 1:** The siRNA oligos used in this study.

siRNA oligo	sense	anti-sense	position
siRNA371	5'-CGCCAAUCGAAAGCGUGAAUCCGAA-3'	5'-UUCGGAUUCACGCUUUCGAUUGGCG-3'	371–395
siRNA643	5'-AAGCGCAUGCCAGAGAGCUUCAGAA-3'	5'-UUCUGAAGCUCUCUGGCAUGCGCUU-3'	643–667
siRNA1743	5'-CAGGCGGAAAUUGCCGAACUGGAAA-3'	5'-UUUCCAGUUCGGCAAUUUCCGCCUG-3'	1,743–1,767
control siRNA	5'-AUCGGCUACCAAGUCAUACACAGUC-3'	5'-GACUGUGUAUGACUUGGUAGCCGAU-3'	

### Synthesis of dsRNAs

Three DNA fragments of *Ts*-*pmy*, based on the sequence regions 75–974 (PF1), 924–1,874 (PF2) and 1,825–2,668 (PF3), were generated with PCR using gene-specific primers flanked by T_7_ RNA polymerase promoter sequences. In addition, a DNA fragment (13–1,056) of *Ts*87, a *T. spiralis-*specific surface antigen [22], was generated with PCR for use as a parallel control.

The PCR fragments were used to generate dsRNAs through *in vitro* transcription using the MEGAscript™RNAi kit (Life Technologies, US). To confirm integrity, single-stranded RNA and dsRNA were visualized on a 1% agarose gel. The concentration of each dsRNA was determined using a NanoDrop 2000 spectrophotometer (Thermo, UK). A 500-bp irrelevant dsRNA from the MEGAscript™RNAi Kit (Life Technologies, US) served as a negative control.

### SiRNA or dsRNA Delivery to *T. spiralis* Worms

*T. spiralis* larvae and adult worms were recovered from the infected ICR mice and washed three times in 0.9% saline solution. For soaking incubation, 2,000 larvae or 500 adult worms were suspended in a final volume of 500 µl culture medium RPMI 1640 (Gibco, HyClone) supplemented with 20% heat-inactivated fetal bovine serum (FBS, Sigma), 100 units/ml penicillin and 100 µg/ml streptomycin. Specific or control siRNA or dsRNA were incubated with 2 µl Lipofectin Reagent (Life Technologies, US) and 0.8 unit RNaseOUT™ (Life Technologies, US) for 10 min before being added to the larvae or adult worms to a final concentration of 4µM for siRNA and 50 ng/µl for dsRNA. The incubation was continued at 37°C and 5% CO_2_ for up to 6 days.

For electroporation, 2,000 larvae or 500 adult worms were suspended in 100 µl of electroporation buffer (Life Technologies, US) containing the same amount of siRNA or dsRNA as used for soaking incubation. The worm suspension was electroporated at 125 V for 20 ms by using a Gene Pulser II System (Bio-Rad, US), then added with culture medium up to 500 µl and incubated at 37°C and 5% CO_2_ for up to 10 days. FITC-labeled control siRNA was used as for visualizing the uptake of siRNA.

Conditions for incubation or electroporation (125 V, 20 ms) have been confirmed to be safe for keeping the worms alive up to 10 days when control siRNA or dsRNA were added.

### Quantitative Real-time PCR (qRT-PCR)

Total RNA was extracted from siRNA- or dsRNA-treated larvae or adult worms using an RNAsimple Total RNA Kit (Tiangen, China) according to the manufacturer’s instructions. The extracted RNA was treated with 200 U DNAseI (Tiangen, China) to remove any genomic DNA contamination. The quality of the RNA was visualized with 1.2% denatured agarose gel electrophoresis using the One-Stop RNA Electrophoresis kit (TIANZE, China). The concentration of RNA was measured with the NanoDrop2000.

For qRT-PCR, 50 ng of total RNA from treated worms was reverse transcribed to first-strand cDNA using the Sensiscript® reverse transcription kit (Qiagen, US) and oligo dT_15_ primer. The following primers were designed for qRT-PCR: *Ts*-*pmy* (forward: CAG TCG GAA GTT GAA GTT TTG; reverse: CTC TGA GTT CTC G TT GTT GCG TAG); *Ts*87 (forward: GCA ACA GTA CCC GCT TTC TAT GAT TCA AC; reverse: TCA AAG TCG CTA CGC CAT CAC CAG); and GAPDH, which served as the endogenous control (forward: TGC TTC TTG CAC TAC CAA TGG CTT AG; reverse: ACC AGA TGG ACC ATC GAC TGT CTT TT). The qRT-PCR was conducted in triplicate using the Brilliant® II SYBR® Green QPCR Master Mix kit (Stratagene, US) and the Chromo4™ real-time PCR Detector (Bio-Rad, US) to evaluate target gene expression. Reactions were performed with 40 cycles of 1 min at 95°C, 20 s at 60°C and 20 s at 72°C. The levels of *Ts-pmy* transcripts in siRNA- or dsRNA-treated worms were calculated as the percentage relative to the level of untreated worms by using double standard curves calibrated with plasmid DNA containing *Ts-pmy* and endogenous control DAPDH gene, according to manufacturer’s protocol. Standard curves had a correlation coefficiency R^2^>0.99. QPCR data were analyzed with Bio-Rad Opticon Monitor™ software.

### Evaluation of Protein Expression

SiRNA- or dsRNA-treated worms were harvested 6 days post-soaking incubation or electroporation. The harvested worms were homogenized to prepare the crude somatic extracts. The total protein concentrations were determined with a BCA assay (Pierce, US). An equal amount of protein from each treated worm group was separated through SDS-PAGE and subsequently transferred onto nitrocellulose membranes. The membranes were blocked with 5% (w/v) nonfat dry milk in Tris-buffered saline with 0.05% Tween 20 (TBS-T) and incubated with mouse antisera against *Ts*-PMY (1∶10,000) or *Ts*87 (1∶100) [17]. In addition, rabbit anti-GAPDH (1∶1,000, Cell Signaling Technology, US) was used to detect GAPDH expression as a quantitative protein control. IRDye 800CW-conjugated goat anti-mouse or rabbit IgG (H+L) (LI-COR, US) was used as secondary antibodies, and the reactions were detected using the Odyssey detection system (LI-COR, US).

### Viability of siRNA-treated *T. spiralis* Worms

To observe the effect of RNAi on the molting and viability of treated larvae, the larvae were electroporated with siRNA1743 or control siRNA. Since the larva immediately invades intestinal mucosa and begins to molt 8–14 hours post tissue invasion during the natural infection [23], we started to count the molting larvae 18 hours after being electroporated with siRNA1743. Only those with apparent cuticle sheath at one or two ends of larva were counted as molting larvae. To measure the viability of the siRNA-treated larvae, 2 µM SYTOX green dye was added to the treated larvae. Larvae with low viability or surface damage were stained with fluorescence [24]–[26]. After incubation for 15 min, the larvae were examined under a fluorescence dissecting microscope to determine the percentage of larvae that incorporated the fluorescence. The fluorescence density of siRNA-treated larvae was measured with a Fluoroskan Ascent fluorometer (Thermo Labsystems, US) at 485 nm excitation and 530 nm emission. The fluorescent ratios to the worms treated with control siRNA were calculated as the average of triplicate values.

To examine the infectivity of siRNA-treated larvae, 60 mice were equally divided into 3 groups. Each group was orally infected with 500 *T. spiralis* larvae electroporated with siRNA1743, control siRNA or medium only within 3 hours. Ten mice from each group were sacrificed 4 days post infection, and the adult worms were collected from the intestine of sacrificed mice and counted. The fecundity of recovered female worms was observed after being incubated individually in each well of 24-well tissue culture plate with culture medium for 3 days, and the number of newborn larvae produced by each female worm was counted. The muscle larvae were collected from the remaining 10 mice of each group 35 days post infection using a routine digestion method described previously [20]. The worm reduction was calculated based on the mean number of adult worms or muscle larvae collected from the group treated with siRNA1743 compared with the control siRNA-treated group.

### Statistics

Data were expressed as the mean ± standard error (S.E.) and evaluated by post-hoc test in ANOVA. *p*<0.05 was regarded as statistically significant. For all experiments, the gene expression level was set to 100% in untreated group as standard. The statistical difference in treated group was obtained by comparing with the group treated with control siRNA or dsRNA.

## Results

### SiRNA-mediated Suppression of *Ts-pmy* mRNA Expression

**Figure 1:**
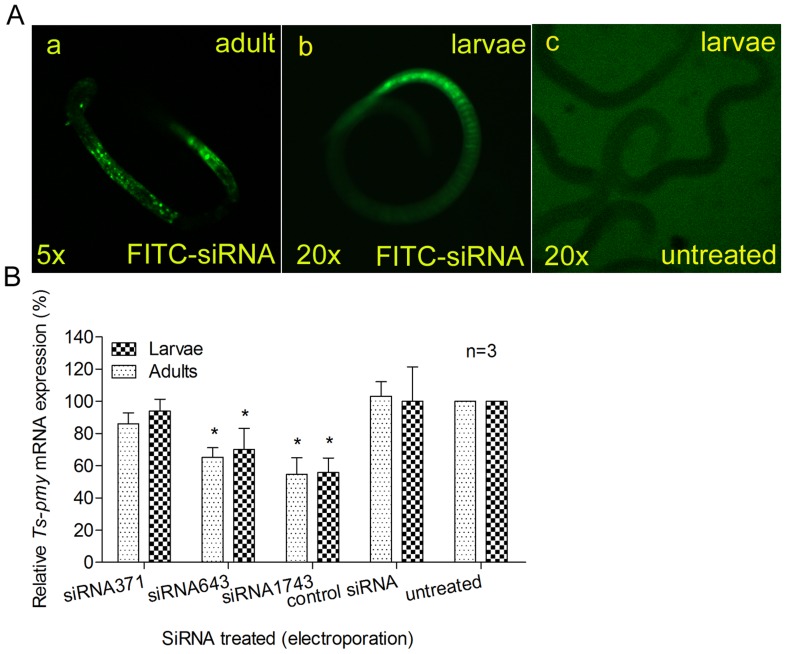
The silencing of *Ts-pmy* mRNA mediated by siRNA. The adult and larval *T. spiralis* were transfected with FITC-labeled control siRNA by electroporation (A). Uptake of FITC-labeled siRNA into adult worms (a) and larvae (b) 18 hours after electroporation under a fluorescent microscope. No fluorescence was observed in the untreated group (c). The relative levels of *Ts-pmy* mRNA in parasites (larvae and adults) 6 days after being electroporated with different siRNAs, as determined with qRT-PCR (B). All of the assays were performed in triplicate, and the data are presented as the mean ± SE. *p<0.05 compared with control siRNA.

Eighteen hours after being electroporated with FITC-labeled control siRNA, more than 75% of the treated worms (larvae and adults) displayed fluorescence staining in different tissues under fluorescent microscopy (Fig. 1A). After being soaked with FITC-labeled siRNA for 60 hours, more than 70% of the worms (larvae and adults) also showed significant uptake of fluorescence (data not shown), indicating that siRNA can be efficiently delivered into *T. spiralis* worms through electroporation or soaking.

After being electroporated with 4 µM of siRNA643 or siRNA1743 for 6 days, the *T. spiralis* larvae or adult worms expressed significantly reduced level of *Ts-pmy* mRNA compared with worms treated with control siRNA or untreated worms (Fig. 1B). The relative levels of *Ts-pmy* mRNA expression in adult worms treated with siRNA643 or siRNA1743 detected by qRT-PCR were 65.3% and 54.6% of the level in control siRNA-treated worms, respectively (*p*<0.05). SiRNA371 did not elicit a significant reduction in the expression of *Ts-pmy* mRNA. There was no significant difference in silencing efficiency between larvae and adult worms. The control siRNA had no inhibitory effect on *Ts*-*pmy* mRNA expression.

**Figure 2:**
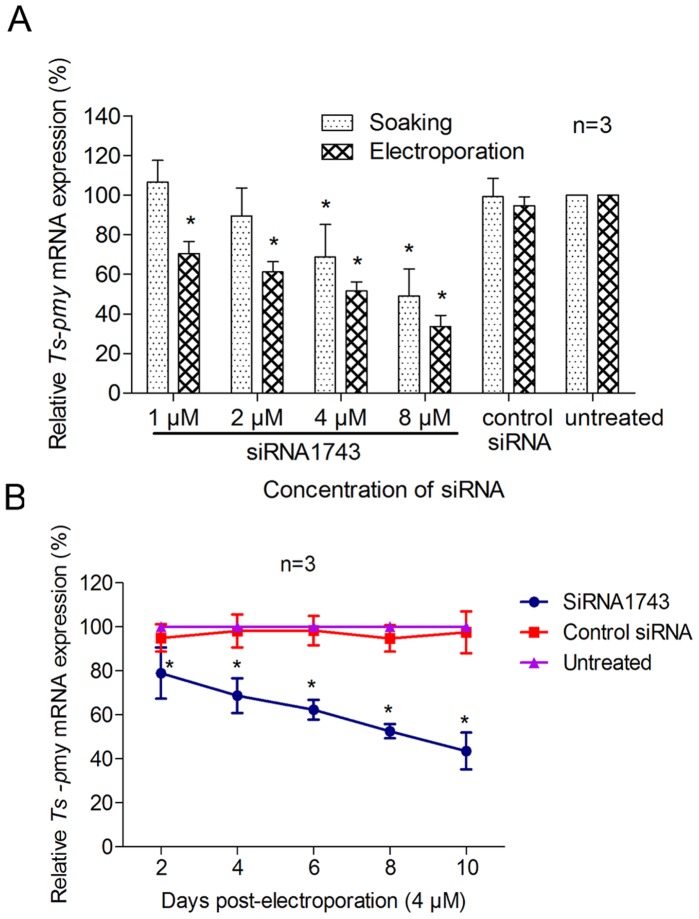
The silencing of *Ts-pmy* mRNA in larvae treated with siRNA1743. The relative level of *Ts-pmy* mRNA in larvae following electroporation or soaking with various concentrations of siRNA1743 for 6 days, as determined with qRT-PCR (A). The better siRNA suppressive effect in treated larvae was observed when a longer incubation was applied after electroporation (B). All of the assays were performed in triplicate, and the data are presented as the mean ± SE. *p<0.05 compared with control siRNA.

Because siRNA1743 produced the highest silencing of *Ts-pmy* mRNA expression, it was used to optimize the working concentration and delivery approach in larva transfection. The efficacy of siRNA silencing was dose dependent, and the most effective silencing was observed at 8 µM which reduced the expression of *Ts-pmy* mRNA in treated larvae to 33.7% of the level in control siRNA-treated worms (66.3% reduction) when delivered with electroporation for 6 days (Fig. 2A). Even though the silencing efficiency induced by electroporation looked better than that induced by soaking, the difference was not statistically significant.

A longer incubation of worms after electroporation with siRNA1743 led to better silencing efficiency of *Ts*-*pmy* mRNA expression (Fig. 2B). Ten days after 4 µM of siRNA1743 was delivered with electroporation, the expression of *Ts*-*pmy* mRNA was 43.5% of the levels in control siRNA-treated larvae.

### DsRNA-mediated Suppression of *Ts-pmy* mRNA Expression

**Figure 3:**
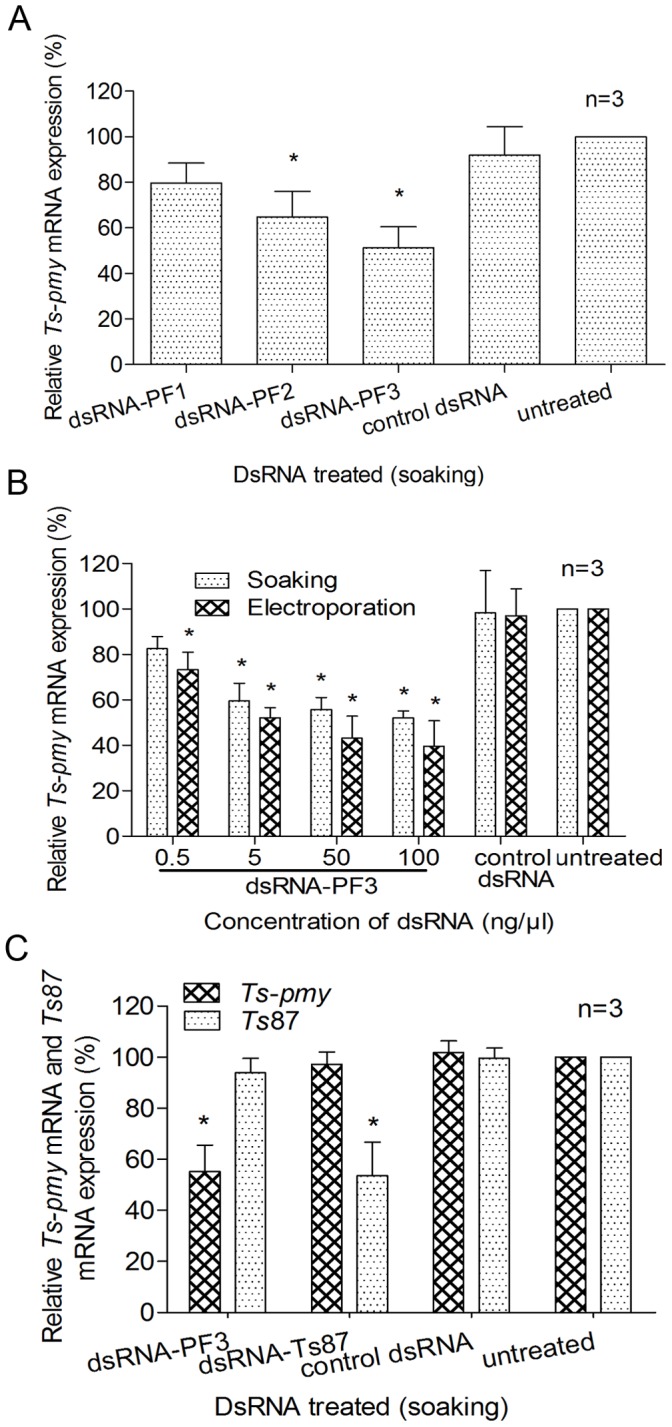
The silencing of *Ts-pmy* mRNA mediated by dsRNA. The relative level of *Ts-pmy* mRNA expression in larvae soaked with different dsRNAs (50 ng/µl) for 6 days (A). The relative level of *Ts-pmy* mRNA in larvae following electroporation or soaking with various concentrations of dsRNA-PF3 for 6 days, as measured with qRT-PCR (B). Specific suppression of *Ts-pmy* and *Ts*87 mRNA expression in larvae 6 days after being soaked with 50 ng/µl dsRNA-PF3 or *Ts*87 dsRNA (C). All of the assays were performed in triplicate, and the data are presented as the mean ± SE. *p<0.05 compared with the control dsRNA-treated group.

Similar to siRNA, the best *Ts-pmy* mRNA silencing was obtained in larvae transfected with dsRNAs derived from the C-terminus of *Ts*-*pmy* (dsRNA-PF3). The relative levels of *Ts-pmy* mRNA expression in parasites soaked with dsRNA-PF2 and dsRNA-PF3 for 6 days were only 64.7% and 51.2% of levels in the control dsRNA-treated group (Fig. 3A). There was no significant decrease in the level of *Ts-pmy* mRNA expression in the larvae treated with dsRNA-PF1. The control dsRNA had no inhibitory effect on *Ts-pmy* mRNA expression.

The dsRNA-mediated inhibition of *Ts-pmy* mRNA expression occurred in a dose-dependent manner for dsRNA-PF3 (Fig. 3B). The best *Ts-pmy* silencing was achieved 6 days after *T. spiralis* larvae were electroporated with 100 ng/µl of dsRNA-PF3 (60.4% reduction). Electroporation had a higher efficiency than soaking in inhibiting the expression of the target gene, but the difference was not statistically significant, as observed for siRNA. The similar silencing efficiency for *Ts-pmy* mRNA transcription was observed in worms induced by dsRNA-PF3 and siRNA1743 (60.4% and 66.3% reduction, respectively).

The dsRNA-mediated silencing of *Ts-pmy* mRNA expression was gene specific. *Ts-pmy* dsRNA-PF3 only silenced *Ts-pmy* mRNA expression, whereas the *Ts*87 dsRNA knocked down the expression of *Ts*87 mRNA (Fig. 3C).

### Reduction of *Ts*-PMY Protein Expression in Parasites Treated with *Ts-pmy* siRNA or dsRNA

**Figure 4:**
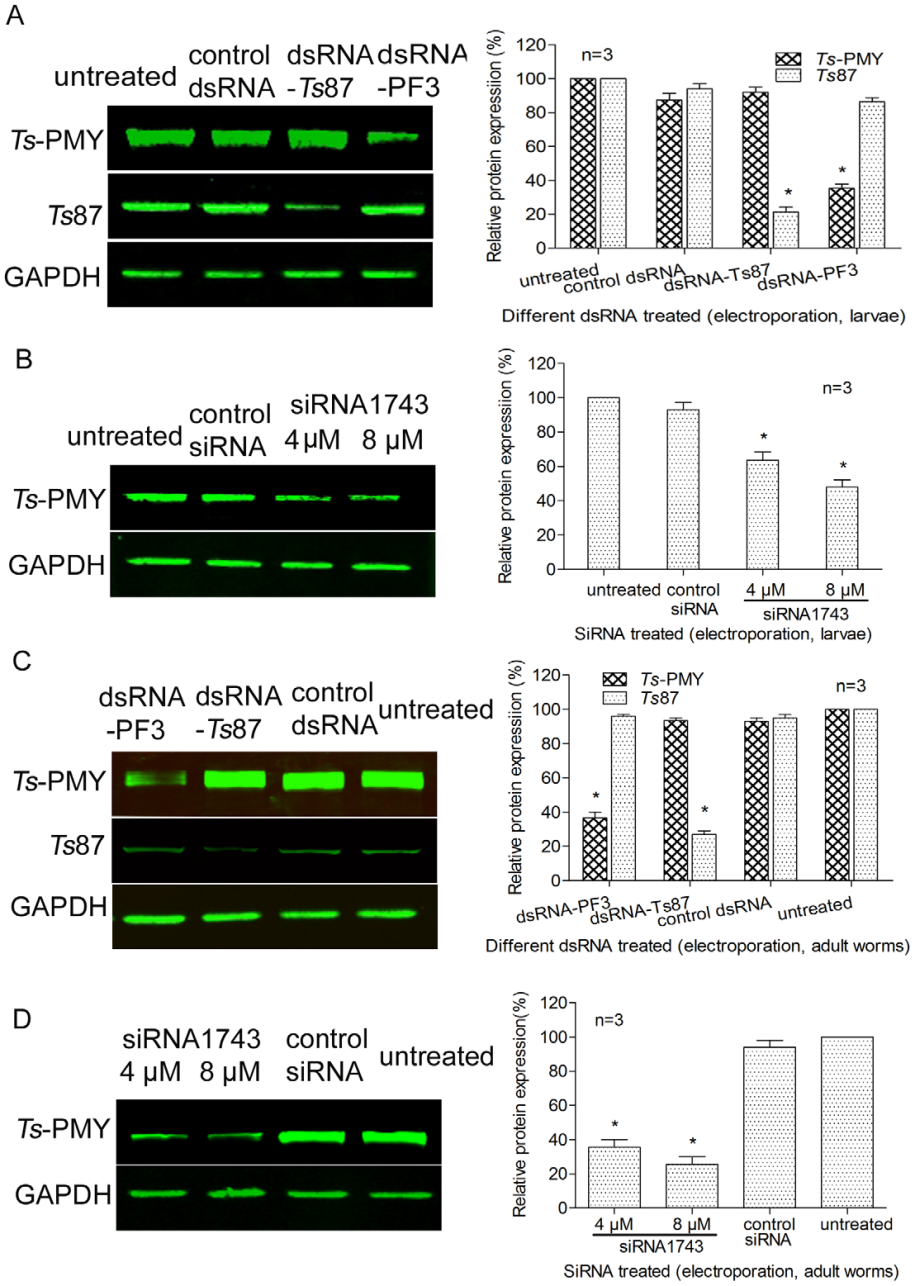
The silencing of *Ts-*PMY protein expression mediated by siRNA or dsRNA. Western blot with specific antibodies showing the specific inhibition of *Ts*-PMY protein expression in extracts of *T. spiralis* larvae (A, B) and adult worms (C, D) induced by dsRNA-PF3 (A, C) or siRNA1743 (B, D). Graphs on the right show the relative protein levels measured by densitometry from three independent experiments. *p<0.05 compared with the control dsRNA/siRNA-treated group.

To determine whether the silencing of *Ts*-*pmy* mRNA mediated by the siRNA or dsRNA reduces the level of *Ts*-PMY protein expression, Western blotting with specific antibodies was performed on the extracts of treated adult worms and larvae. The expression of the *Ts*-PMY protein was significantly decreased in both adult worms and larvae treated with either siRNA1743 or dsRNA-PF3 compared with worms treated with control siRNA or dsRNA (Fig. 4). The expression of the *Ts*-PMY protein was inhibited 52.0% and 74.5% when larvae and adult worms were electroporated with 8 µM of siRNA1743, respectively, compared with control siRNA treated worms (Fig. 4B and 4D). The *Ts*-PMY protein expression level reduced 64.7% and 63.5% in larvae and adult worms, respectively, after being eletroporated with 100 ng/µl of dsRNA-PF3 compared to the control dsRNA group (Fig. 4A and 4C). In control worms treated with *Ts*87 dsRNA, only the expression of the *Ts*87 protein was suppressed (Fig. 4A and 4C). The housekeeping protein GAPDH was detected in all of the worm extracts at similar levels and was not affected by any *Ts-pmy* or *Ts*87 siRNA or dsRNA treatment.

### Suppression of *Ts*-*pmy* mRNA Impaired the Molting and Viability of *T. spiralis* Larvae

**Figure 5:**
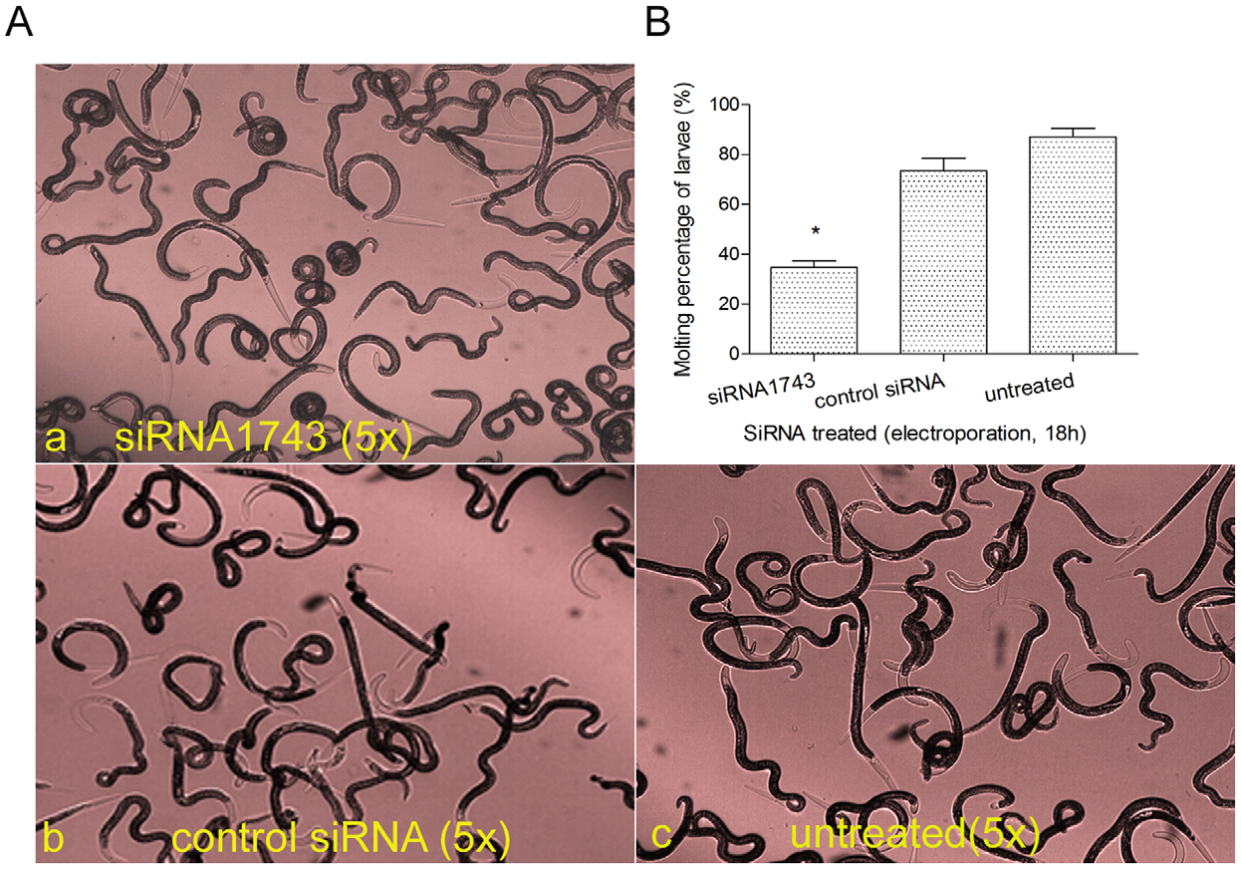
Suppression of *Ts*-*pmy* mRNA expression by siRNA1743 caused defects in larval molting. A. The larvae were electroporated with 8 µM siRNA1743 (a) or control siRNA (b) and incubated for 18 hours. The untreated larvae (c) were used as a negative control. The larvae with cuticle sheath at the end(s) were counted as molting ones. B. The molting percentage for each treatment was calculated. All of the assays were performed in triplicate. *p<0.05 compared with the control siRNA-treated group.

Knockdown of *Ts-pmy* mRNA by RNAi resulted in defect in *T. spiralis* larval molting. The molting rate was only 32.7% for larvae eletroporated with 8 µM siRNA1743 for 18 hours while 73.5% and 87.1% of larvae treated with control siRNA and untreated occurred molting (Fig. 5). The difference of molting rate between siRNA1743-treated group and control siRNA-treated group was statistically significant (40.8% reduction), indicating the reduction in larval molting rate was caused by specific inhibition of *Ts-pmy* mRNA expression. There was no significant reduction of molting rate in larvae electroporated with control siRNA and untreated larvae, suggesting the electroporation did not significantly cause the defect in larval molting. Our results suggested that PMY may play a role in regulating molting during larval development. Some of the normal larvae (untreated or treated with control siRNA) did not show the molting probably because the culture condition *in vitro* may not completely reflect the condition *in vivo*.

**Figure 6:**
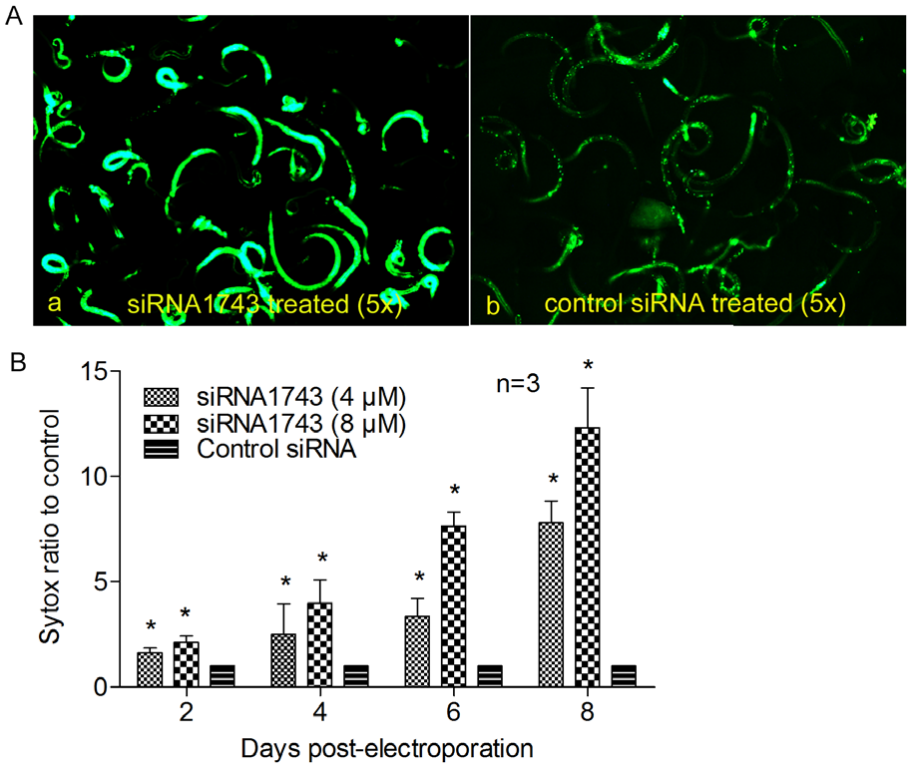
Suppression of *Ts*-*pmy* mRNA expression by siRNA1743 caused surface damage and defected viability of treated larvae. The larvae treated with siRNA1743 for 6 day were stained with SYTOX for 15 min and then observed under fluorescence microscopy (A) or measured with a fluorescence microplate reader at 485 nm excitation and 530 nm emission (B). All of the assays were performed in triplicate. *p<0.05 compared with the control siRNA-treated group.

The suppression of *Ts-pmy* mRNA expression also resulted in larval surface damage and defect in larval viability. The larvae electroporated with siRNA1743 for 6 days showed more larvae stretched out from coiled form and less motile compared with those treated with control siRNA under microscopic observation (Data not shown). The siRNA1743 treated larvae were also incorporated with more fluorescence after being stained with SYTOX compared with those treated with control siRNA (Fig. 6A), indicating the surface damage and the defected viability in larvae treated with siRNA1743. Higher concentrations of siRNA1743 or longer the treatment time caused more serious surface damage and defected viability in treated larvae, indicating the damage is correlated to the suppressive level of *Ts-pmy* mRNA expression as showed in Fig. 2A and 2B. The larvae treated with 8 µM siRNA1743 for 8 days had incorporated more than 10 times higher fluorescence than those treated with control siRNA, as measured with a fluorescence microplate reader (Fig. 6B). The larvae treated with control siRNA showed much less fluorescent binding, suggesting the larval damage and the viability defect was siRNA1743-specific and not much related with electroporation.

### Suppression of *Ts-pmy* mRNA in *T. spiralis* Larvae Affects Parasite Survival *in vivo*

Mice infected with *T. spiralis* larvae transfected with siRNA1743 by electroporation displayed a 37.6% reduction in adult worm burden and 23.2% reduction in muscle larval burden compared with the groups infected with control siRNA-treated larvae, and these differences were statistically significant (Table 2) (*p*<0.05).

**Table 2:** Recovery of adult worms, muscle larvae and newborn larvae of *T. spiralis* from mice infected with larvae transfected with siRNA1743 by electroporation.

Group	Adult worms recovered (mean± S.E)	Reduction (%)	Muscle Larvae recovered (mean± S.E)	Reduction (%)	Newborn larvae/female (mean± S.E)	Reduction (%)
siRNA1743	123.6±27.3	37.6*	34,885±3262	23.2*	75.8±18.8	24.8*
control siRNA	197.9±17.1		45,420±5173		100.6±24.5	
untreated	201.9±32.0		46,675±5270		104.5±20.8	

* *p*<0.05 compared with the control siRNA-treated group.

### Suppression of *Ts-pmy* mRNA Reduced Female Parasite Fecundity

The female adult worms collected from mice infected with siRNA1743-treated larvae demonstrated a significant reduction (24.8%) in producing newborn larvae compared with control siRNA-treated groups after being cultured *in vitro* for 72 hours (Table 2), but there was no significant difference in the motility of the newborn larvae compared with the control group (data not shown).

## Discussion

Here we report the successful silencing of *Ts*-PMY in the larvae and adult worms of *T. spiralis* at either mRNA transcription or protein expression levels induced by specific siRNA and dsRNA. The knockdown of *Ts*-PMY expression in *T. spiralis* larvae resulted in impaired molting and viability *in vitro* and significant lower infectivity in mice, indicating the *Ts*-PMY plays an important role in the viability and survival of *T. spiralis* in the host. Specific gene silencing by RNA interference (RNAi) was first established in *Caenorhabditis elegans* and developed in this organism for high-throughput functional genomics analysis [27]–[29]. With the ease and reproducibility of RNAi in *C. elegans* and the conservation of RNAi mechanisms in a wide variety of other organisms [27], [30], this method has swept through all fields of eukaryotic biology, from yeasts and plants to animals, especially when organisms are not amenable to classical genetic approaches, with the aim to directly assess gene functions [31]–[34]. In parasitic nematodes, *Nippostrongylus brasiliensis* was the first animal parasitic nematode in which RNAi was successfully performed [35]. Subsequently, RNAi has been widely used in several parasitic nematodes, including *Brugia malayi* [36]–[37], *Onchocerca volvulus* [38], *Ascaris suum* [39], *Trichostrongylus colubriformis* [40], *Litomosolides sigmodontis* [41], *Haemonchus contortus* [42]–[45] and *Heligmosomoides polygyrus* [46], with a reduction in gene expression and visible suppressed phenotypes although they appear variable. There is no published report on using RNAi to study gene function in *T. spiralis* up to date although it has become a fascinating model organism for immunologists and biochemists [10]. Furthermore, the entire genomic sequencing of *Trichinella* has been completed, but the functions of many genes are unclear [47]. The development of RNAi technology in *T. spiralis* would allow us to exploit the data through manipulation of the *Trichinella* genome and provide a more direct approach for identifying essential genes and their products as targets for vaccine and drug development against trichinellosis. In this paper, for the first time, we report the significantly silenced transcription of *Ts-pmy* mRNA and expression of *Ts*-PMY protein in larval and adult *T. spiralis* through both siRNA and dsRNA. Both soaking and electroporation effectively delivered the siRNA or dsRNA into the worms. Using fluorescence-labeled siRNA as a control, we observed fluorescence distributed in different parts and organs of the treated worms (Fig. 1A), indicating that siRNA could penetrate the parasite through not only the mouth or excretory openings but also the surface cuticle or hypodermis of larvae and adult worms. It also could be that siRNA is entering through a single point source e.g. gut, and then spreading to these locations through mechanisms involving SID/RSD homologues [48]–[50].

Different delivery methods of RNAi can achieve various silencing results and phenotypes [46], [49]. In this study, siRNA or dsRNA delivered by electroporation had better silencing efficiency for *Ts-pmy* expression than those delivered by soaking in both larvae and adult worms, possibly because the electroporation shock may temporarily open channels on the surface of worms (cuticle or hypodermis) or mouth/excretory opening or mediate direct transmembrane transit of more siRNA or dsRNA to enter the worm tissues, therefore is not only dependent on SID-like uptake transporters [40], [51]–[52]. However, the combination of electroporation and soaking did not increase *Ts-pmy* silencing level induced by siRNA (data not shown), as shown by Pierson et al in *Moniezia expansa* [53], possibly because of the quick degradation of siRNA in the medium. Many results have confirmed that more effective gene suppression can be achieved when dsRNA is used in nematodes [27], [51], [54]. In our study, both dsRNA and siRNA had similar silencing effects on the expression of *Ts*-*pmy* mRNA or protein (Figs. 1, 3, 4).

The impaired viability of *T. spiralis* larvae with inhibited *Ts-pmy* mRNA and protein expression induced by RNAi was observed through *in vitro* culture and *in vivo* infection in mice. Silencing *Ts-pmy* mRNA and *Ts*-PMY protein expression caused deficit in *T. spiralis* larval molting, the necessary process for larval development, indicating the PMY may be involved in the development of *T. spiralis* larva. It was also observed that *T. spiralis* larvae stretched out from their coiled form and were less motile after being treated with *Ts-pmy* siRNA compared with the group treated with irrelevant control siRNA. Fluorescent SYTOX staining showed that *Ts-pmy* siRNA-treated larvae were stained with more fluorescence, indicating more surface damage and lower viability. This is the first time for SYTOX green being used to determine the surface damage and viability of *T. spiralis* larvae although it has been used for other nematodes, such as *H. glycines* and *C. elegans* [25]–[26], [55]. These impaired viability observations are partially consistent with the report of RNAi induced PMY suppression in *C. elegans*, in which some phenotypes such as uncoordinated, paralyzed worms and egg laying defects were observed in the RNAi treated free-living nematode [24]. The *in vivo* infection of mice with larvae transfected with *Ts-pmy* siRNA demonstrated a 37.6% reduction in adult burden and 23.2% reduction in muscle larvae burden compared with mice infected with control siRNA-treated larvae. In addition, adult worms recovered from mice infected with siRNA-treated larvae released 24.8% less newborn larvae.

These impaired viabilities are totally related to the specific silencing of *Ts-pmy* mRNA or protein expression because the control siRNA did not exhibit significant reduction in larval viability compared to untreated larvae. The condition for electroporation (125 V, 20 ms) has been confirmed not to impair the viability of treated larvae or adult worms up to 10 days. All of the results demonstrated that silencing PMY expression in *T. spiralis* significantly reduced parasite viability and infectivity, further indicating that *Ts*-PMY plays important roles in the life cycle of *T. spiralis* and survival in host. The expression of PMY in *T. spiralis* is necessary, at least for the development of larvae and fecundity of female worms, in addition to its function as an immunomodulatory protein against host complement attack [17]–[18], therefore confirming its promising target for vaccine development.
